# Identifying Hierarchical and Overlapping Protein Complexes Based on Essential Protein-Protein Interactions and “Seed-Expanding” Method

**DOI:** 10.1155/2014/838714

**Published:** 2014-06-30

**Authors:** Jun Ren, Wei Zhou, Jianxin Wang

**Affiliations:** ^1^College of Information Science and Technology, Hunan Agricultural University, Changsha 410128, China; ^2^School of Information Science and Engineering, Central South University, Changsha 410083, China; ^3^Hunan Provincial Key Laboratory of Crop Germplasm Innovation and Utilization, Changsha 410128, China

## Abstract

Many evidences have demonstrated that protein complexes are overlapping and hierarchically organized in PPI networks. Meanwhile, the large size of PPI network wants complex detection methods have low time complexity. Up to now, few methods can identify overlapping and hierarchical protein complexes in a PPI network quickly. In this paper, a novel method, called MCSE, is proposed based on *λ*-module and “seed-expanding.” First, it chooses seeds as essential PPIs or edges with high edge clustering values. Then, it identifies protein complexes by expanding each seed to a *λ*-module. MCSE is suitable for large PPI networks because of its low time complexity. MCSE can identify overlapping protein complexes naturally because a protein can be visited by different seeds. MCSE uses the parameter *λ*_th to control the range of seed expanding and can detect a hierarchical organization of protein complexes by tuning the value of *λ*_th. Experimental results of *S. cerevisiae* show that this hierarchical organization is similar to that of known complexes in MIPS database. The experimental results also show that MCSE outperforms other previous competing algorithms, such as CPM, CMC, Core-Attachment, Dpclus, HC-PIN, MCL, and NFC, in terms of the functional enrichment and matching with known protein complexes.

## 1. Introduction

High-throughput techniques, such as yeast-two-hybrid [1], mass spectrometry [2], and protein chip technologies [3], have led to the emergence of large protein-protein interaction (PPI) data sets. Such PPI data can be downloaded easily from public biological databases such as DIP [4], MIPS [5], and SGD [6]. They are naturally represented in the form of networks, where vertices are proteins and edges are protein interactions. As many evidences have indicated that PPI network is a “small-world” network [7, 8] and dense subgraphs or modules in it generally correspond to protein complexes [9–13], a series of clustering methods are proposed to identify protein complexes in PPI network [12–31].

The most popular methods are density-based methods, such as CPM [15, 16], CMC [17], Core-Attachment [18], Dpclus [19], and IPCA [20]. They identify protein complexes as dense subgraphs in PPI networks and usually have good performance because dense subgraphs in PPI networks generally correspond to protein complexes. Meanwhile, they can identify overlapping protein complexes naturally because dense subgraphs are overlapping. The main disadvantage of them is that they cannot detect the hierarchical organization of protein complexes. However, protein complexes in biological organisms are hierarchically organized [32–36]. For example, the GO annotation in GO database [32] and SGD database [36] are hierarchically organized. A more direct example is the hierarchical structure of known protein complexes of* S. cerevisiae* listed in the MIPS database [34].

To detect the hierarchical organization of protein complexes, hierarchical clustering algorithms, such as Monet [13] and HC-PIN [21], are proposed. They start from a partition in which each node is its own community and merge clusters according to a topological measure of similarity between nodes. These methods can identify hierarchical organization of protein complexes naturally, but they cannot identify overlapping protein complexes because the initial clusters are unoverlapping nodes and the merging process cannot produce overlapping. However, many evidences have demonstrated that a protein can be in several protein complexes.

To identify both overlapping and hierarchical protein complexes in PPI network, algorithms proposed to detect the overlapping and hierarchical communities in complex networks, such as EAGLE [37] and NFC [38], can be used in PPI network. However, they both have limitations. EAGLE has high time complexity and is not suitable for large PPI networks [37]. NFC is a “seed-expanding” method and its seeds are selected randomly, which may results in the poor performance for detecting protein complexes [27, 38].

To identify both overlapping and hierarchical protein complexes in PPI network accurately and fast in large PPI networks, a novel algorithm, namely MCSE, is proposed based on “seed-expanding.” It first builds a weighted PPI network from the input PPI network according to edge clustering value. Then, it chooses essential PPIs and PPIs whose edge weights are more than average weight as seeds. At last, it identifies protein complexes by expanding each seed to a *λ*-module in the weighted PPI network. MCSE runs fast and identifies overlapping protein complexes naturally because it is a “seed-expanding” method. The construction of weighted PPI network and the selection of seed in MCSE improve its efficiency. MCSE uses the parameter *λ*_th to control the expanding range and can detect protein complexes in different hierarchical levels by tuning *λ*_th value. Experimental results of* S. cerevisiae* show that the hierarchical structure of protein complexes identified by MCSE is approximately corresponding to that of known protein complexes in MIPS database. More importantly, MCSE can identify protein complexes more accurately than other competing algorithms, such as CPM [15, 16], CMC [17], Core-Attachment [18], Dpclus [19], HC-PIN [21], MCL [31], and NFC [38].

## 2. Methods

To identify both overlapping and hierarchical protein complexes in PPI networks fast, we develop a novel protein complex detection method based on “seed-expanding”. Seed-expanding method is a local search method and has low time complexity. It can identify overlapping protein complexes naturally because a protein can be visited by different seeds. To develop a seed-expanding method, three issues should be solved: (1) seed selection; (2) rules for expanding, which decide which node can be added into the expanding cluster; (3) finish conditions, which decide the end of an expansion from a seed. We explicate these three issues of our method as follows.

### 2.1. Seed Selecting

**Figure 1:**
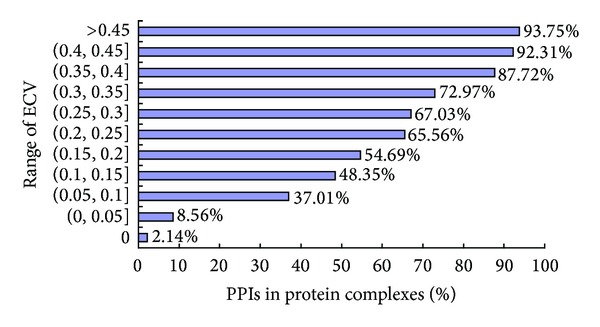
The percentage of PPIs in protein complexes with respect to different range of edge clustering value (ECV).

Many evidences have indicated that a PPI with high edge clustering value in a PPI network has high possibility to be in a protein complex [21, 24]. To verify whether it is true or not, Figure 1 shows the percentage of PPIs in protein complexes with respect to different range of edge clustering value (ECV). The PPI network is a PPI network of* S. cerevisiae* downloaded from DIP database (version 2010.6 http://dip.doe-mbi.ucla.edu/dip/Download.cgi/) and named as YDIP [39]. The protein complex set of* S. cerevisiae *is the latest one provided by Pu et al. in [40], which includes 408 complexes and is named as CY408. The edge clustering value of an edge 〈*u*, *v*〉, namely, ECV(*u*, *v*), in a PPI network *G* is calculated as [21],

(1) $$\begin{array}{c} \text{ECV}\left(u,v\right)=\frac{\sum_{k\in I_{u,v}}w_{u,k}*\sum_{k\in I_{u,v}}w_{v,k}}{\sum_{s\in N_{u}}w_{u,s}*\sum_{t\in N_{v}}w_{v,t}}|, \end{array}$$

where *w*_*u*,*k*_ is the weight of edge 〈*u*, *k*〉 when *G* is a weighted PPI network and is equal to 1 when *G* is an unweighted PPI network, the *N*_*u*_ and *N*_*v*_ are the sets of neighbors of vertex *u* and vertex *v*, respectively, and *I*_*u*,*v*_ denotes the set of common vertices in *N*_*u*_ and *N*_*v*_ (i.e., *I*_*u*,*v*_ = *N*_*u*_∩*N*_*v*_).

As shown in Figure 1, it is obviously the PPI with high edge clustering value has high possibility to be in a protein complex. So, it is naturally to choose seeds as PPIs with high edge clustering value in a PPI network. According to Figure 1, we simply define the edge's weight as an increasing function of its edge clustering value. The weight of an edge 〈*u*, *v*〉 in a PPI network *G*, namely, *w*(*u*, *v*), is calculated as follows [24]:

(2) $$\begin{array}{c} w\left(u,v\right)=\alpha +\frac{1-\alpha }{{\text{ECV}}_{\text{avg}}}*\text{ECV}\left(u,v\right), \end{array}$$

where ECV_avg_ is the average edge clustering value of the whole PPI network *G*, *α* is a given small constant reflecting the possibility of the PPI with ECV = 0 in a protein complex. Its typical value is 0.2 [24].

According to formula (2), a weighted PPI network is created from the unweighted PPI network* YDIP*. This weighted PPI network has 15,166 PPIs and 2921 (19%) PPIs have weights not less than the average weight. Out of all 15,166 PPIs, only 2130 (14%) PPIs are in protein complexes of* CY408*. Meanwhile, out of 2921 PPIs whose weights are not less than the average weight, 1495 (51%) PPIs are in protein complexes of* CY408*. The possibility of a PPI whose weight is not less than the average weight to be in a protein complex is about 3.64 times of that of a PPI selected randomly. So, it is reasonable to choose PPIs with weight not less than the average weight as seed edges.

Besides topological properties of PPI network, other biological properties are also important prediction factors for protein complexes. For example, He and Zhang classified PPIs as essential PPIs and nonessential PPIs [41]. In recent years, many computational methods have been proposed to identify essential proteins in PPI networks [42–49]. An essential PPI is a PPI whose two proteins are both essential proteins [41]. Essential PPIs are more important than nonessential PPIs because they play more important role in the survival and propagation of living organisms [41]. Thus, it is reasonable to believe that an essential PPI is more likely to be in a protein complex than a nonessential PPI. For example, in all 3045 essential PPIs in the PPI network* YDIP*, 960 (31.5%) are in protein complexes of* CY408*, which is 2.24 times of that of a PPI selected randomly. So, it is reasonable to choose essential PPIs as seed edges.

The two kinds of seed edges are different. One is based on PPI's essentiality. The other is based on PPI's edge clustering value. They complement each other well. So, we assign the final seed set as the union set of both kinds of seed edges. We sort the seed edges by weight first, and by essentiality second, because the PPI with high weight is more likely (the possibility is 51%) to be in a protein complex than an essential PPI (the possibility is only 31.5%).

### 2.2. Rules for Expanding

To decide which node can be added into the expanding cluster, we define the cluster property of a node *v* to a cluster *H*(*v* ∉ *H*) to describe how compactly connected they are. Obviously, the more edges node *v* has to connect to the cluster *H*, the more compactly connected they are, and the more likely they are to belong to the same protein complex. So, in an un-weighted PPI network, it is reasonable to define the cluster property of a node *v* to a cluster *H*(*v* ∉ *H*) as the number of edges connecting *v* and *H*. When a cluster *H* is expanding, the node which has the highest value of cluster property to it will be added into it.

In our method, we first build a weighted PPI network to select seeds. In the weighted PPI network, the higher weight an edge has, the more likely it is to be in a protein complex. Evidences have demonstrated that performance of protein complex detection methods can be improved when they are applied to the weighted PPI network whose edge's weight reflects the possibility of the edge in a protein complex [50–52]. So, it is reasonable to identify protein complexes in our weighted PPI network. In the weighted PPI network, if edges connecting a node *v* and a cluster *H*(*v* ∉ *H*) have higher weights, *v* and *H* are more likely to belong to the same protein complex. Based on it, in a weighted PPI network *G*, we extend the definition of cluster property of a node *v* to a cluster *H*(*v* ∉ *H*), namely, *f*(*v*, *H*), as the sum of weights of edges connecting *v* and *H*. Consider

(3) $$\begin{array}{c} f\left(v,H\right)=\sum_{v\notin H,u\in H,\langle u,v\rangle \in E(G)}w_{u,v}, \end{array}$$

where *E*(*G*) is the edge set of *G*, *w*_*u*,*v*_ is the weight of edge 〈*u*, *v*〉. When *G* is an unweighted PPI network, all edges' weights are equal to 1.

### 2.3. Finish Conditions

Many protein complex models, such as dense subgraph, maximum clique [11, 14], *k*-clique-community [15], weak module and strong module [13, 26], and *λ*-module [21], have been proposed for identifying protein complexes in PPI networks. We choose *λ*-module as protein complex model and finish a seed's expansion when the cluster expanding from the seed is a *λ*-module. Wang et al. defined *λ*-module as a subgraph whose *λ* value is not less than the given *λ* threshold [21]. The *λ* value of a subgraph *H* in a PPI network *G*, namely, *λ*_*H*_, is defined as [21]

(4) $$\begin{array}{c} \lambda_{H}=\frac{\sum_{v\in H}d_{w}^{\text{in}}\left(v,H\right)}{\sum_{v\in H}d_{w}^{\text{out}}\left(v,H\right)}, \end{array}$$

where *d*_*w*_^in^(*v*, *H*) is the weighted in-degree of *v* in *H*, which is defined as the sum of weights of edges connecting vertex *v* to other vertices in *H* and *d*_*w*_^out^(*v*, *H*) is the weighted out-degree of *v* in *H*, which is defined as the sum of weights of edges connecting vertex *v* to vertices in *G* − *H*. The reasons that we choose *λ*-module as the protein complex model in our method are listed as follows.

1. In real biological organism, protein complexes frame a hierarchical organization. The larger protein complex is in higher level and includes (fully or partially) smaller protein complexes in lower levels [32–36]. When a seed edge is expanding, it first reaches a subgraph with small *λ* value, that is a *λ* -module to a small *λ* threshold. Then, with the subgraph expanding, it become a subgraph with larger *λ* value, that is a *λ* -module to a larger *λ* threshold. So, when given a smaller *λ* threshold, the expansion from a seed will be ended quickly and generate a smaller subgraph. When given a larger *λ* threshold, the expansion from the same seed will be ended later and generate a larger subgraph which includes that smaller subgraph corresponding to the smaller *λ* threshold. Thus, by tuning the value of *λ* threshold, we can identify protein complexes in different hierarchical levels.
2. With more and more protein complexes being known, researchers found that many protein complexes are not dense subgraphs in PPI networks [12, 13]. So, using dense subgraph or clique as protein complex model has its own limits. The basic idea behind using *λ*-module as protein complex module is that researchers have found that many protein complexes are densely connected within themselves but sparsely connected with the rest of the PPI network [12, 13, 21]. Thus, our method can identify protein complexes with different density by using *λ*-module as protein complex module.

### 2.4. Algorithm MCSE

Based on the decision of seed selection, rules for expanding, and finish conditions, a novel clustering algorithm based on “seed-expanding,” namely, Mining Complexes based on Seed Expanding (MCSE), is proposed to identify overlapping and hierarchical protein complexes in PPI networks. The detailed description of algorithm MCSE is shown in Algorithm 1. The input of algorithm MCSE is a given value of *λ* threshold *λ*_th, a set of essential PPIs *S*, and a PPI network which is described as a simple undirected graph *G*(*V*, *E*, *W*). The input PPI network can be weighted or unweighted PPI network. If it is an unweighted PPI network, all edges' weights are set as 1.

Algorithm MCSE has four stages: weight calculating, seed selecting, seed expanding, and outputting. Firstly, algorithm MCSE calculate each edge's weight *w*(*v*_*i*_, *v*_*j*_) by formula (2) and build the new weighted PPI network *G* ^*W*^(*V*, *E*, *W*). Secondly, edges whose weights not less than average weight and edges in essential PPIs set *S* are selected as seed edges. They are sorted into seed queue *Sq* in nonincreasing order by the weight first and essentiality second. Thirdly, when the seed queue *Sq* is not null, MCSE will always select the first edge in *Sq* as the seed to expand to a *λ*-module by gradually adding neighbor vertex with highest cluster property. The *λ*-module is considered as an identified protein complex and its vertices are marked. Then, edges which include the marked vertices are removed from *Sq*. The seed expanding will stop when the seed queue *Sq* is null. Finally, MCSE outputs all identified protein complexes. To avoid identified protein complexes highly overlapping, the expansion of a seed will be ended and its expanding cluster will be abandoned if the cluster has more than half of vertices in other identified protein complexes.

## 3. Results

To evaluate the performance of our algorithm MCSE, we compare it with seven previous competing algorithms, CPM [15, 16], CMC [17], Core-Attachment [18], Dpclus [19], HC-PIN [21], MCL [31], and NFC [38], for detecting protein complexes in an unweighted PPI network. Our method MCSE and the other seven algorithms except HC-PIN can all identify overlapping protein complexes. HC-PIN, NFC, and our method MCSE can all detect hierarchical organization of protein complexes. Dpclus, NFC, and our method MCSE are all seed-expanding method. In the experiments, the values of the parameters in each algorithm are selected from those recommended by the authors.

In Section 3, the datasets and evaluation methods used in the paper are described first. Then, performance of our method MCSE and the effect of parameter *λ*_th on clustering results are discussed. Thirdly, the comparison of the known hierarchical protein complexes and those identified by MCSE is studied. Fourthly, the comparison of the performance of MCSE and seven other algorithms is studied in terms of matching with the known protein complexes and functional enrichment. Finally, the effect of seed selection and weighted PPI network for identifying protein complexes is discussed.

### 3.1. Datasets and Evaluation Methods

To test the performance of MCSE, we apply MCSE and other seven algorithms to an unweighted PPI network of* S. cerevisiae*. The original network is downloaded from DIP database (version 2010.6 http://dip.doe-mbi.ucla.edu/dip/Download.cgi/). By removing all the self-connecting interactions and repeated interactions, the final network, named* YDIP*, includes 4,746 proteins and 15,166 interactions. To find the essential PPIs, a list of essential proteins is downloaded from MIPS database (http://dip.doe-mbi.ucla.edu/dip/Download.cgi/), which contains 1,285 essential proteins.

Two kinds of known protein complex set are used in the paper. One is composed of hierarchical protein complexes of* S. cerevisiae* and downloaded from MIPS database [34]. These hierarchical protein complexes form a five-layer forest. The first layer is composed of leaf-complexes which have no subcomplexes. The second layer is composed of the father-complexes of protein complexes in the first layer, and so on. In the five layers, the numbers of complexes are 256, 46, 17, 4, and 1, respectively. The complexes in the top three layers are few. So, to judge the performance for identifying complexes in different levels, we compare the identified complex sets only with the first layer and second layers, respectively. The other kind of protein complex set is provided by the literature published in [40]. It is the latest protein complex set of* S. cerevisiae* but cannot be used to estimate the performance for identifying hierarchical complexes because its protein complexes are all leaf-complexes.

Two kinds of criteria are used in the paper to evaluate the performance of algorithms for identifying protein complexes. One is matching the identified protein complex set with the known protein complex set directly. In the criterion, an identified complex Ic and a known complex Kc are considered as a match if their overlapping score OS(Ic, Kc) is not less than a specific threshold. The overlapping score OS(Ic, Kc) is calculated as [12, 19, 21]

(5) $$\begin{array}{c} \text{OS}\left(\text{Ic},\text{Kc}\right)=\frac{{\left|{V_{\text{Ic}}\cap V}_{\text{Kc}}\right|}^{2}}{\left|V_{\text{Ic}}\right|*\left|V_{\text{Kc}}\right|}, \end{array}$$

where |*V*_Ic_| and |*V*_Kc_| are the numbers of proteins in Ic and Kc, respectively. Based on the match of identified complexes and known complexes, three evaluation criteria are used to quantify the quality of protein complex detection methods:

(1) *Specificity (Sp)* is defined as the fraction of identified complexes matched by known complexes among all identified complexes [12, 19].
(2) *Sensitivity (Sn)* is defined as the fraction of known complexes matched by identified complexes among all known complexes [12, 19].
(3) *F-score* combines the sensitivity and specificity scores [21]. It is defined as
  (6) $$\begin{array}{c} F_{score}=\frac{2*\text{Sp}*\text{Sn}I}{\left(\text{Sp}+\text{Sn}\right)}. \end{array}$$

In the three evaluation criteria, sensitivity is susceptible to the number of identified complexes because the number of known complexes matched by identified complexes will increase with the increase of the number of identified complexes. So, it is not used in the paper as numbers of complexes identified by the eight methods are quite different.

The other criterion is the functional enrichment of the identified complexes. In the criterion, the *P* value of a complex with a given GO term is used to estimate whether the proteins in the complex are enriched for the GO term with a statistically significant probability compared to what one would expect by chance. A complex can have various *P* values for various GO terms. In the paper, the *P* value of a complex defaults to its lowest *P* value. For each identified complex, we use the GO AmiGO (http://amigo.geneontology.org/cgi-bin/amigo/go.cgi) to calculate its *P* value. An identified complex with a smaller *P* value indicates that it is accumulated at random with a smaller chance and is more biologically significant than one with a larger *P* value [30].

### 3.2. Identification of Hierarchical and Overlapping Protein Complexes in the PPI Network of* S. cerevisiae*

Parameter *λ*_th is used to control the expand degree. To evaluate the effect of parameter *λ*_th on clustering results, we set the values of parameter *λ*_th as 0.25, 0.5, 1, 2, 4, 8, and 16 and achieve seven different output sets of identified complexes from* YDIP*. Characteristics of these seven output sets, such as the number of complexes, the average size of complexes, the average density and the minimum density of complexes, and overlapping rate of the complex set, are listed in Table 1. The overlapping rate of a complex set Cset, Or_Cset_, is used to evaluate the overlap of all complexes in Cset and defined as follows [53]:

(7) $$\begin{array}{c} \text{O}{\text{r}}_{\text{Cset}}=\sum_{C_{i}\in \text{Cset}}\frac{\left|C_{i}\right|}{\left|\cup C_{i}\right|}, \end{array}$$

where Cset is a complex set, |*C*_*i*_| is the number of vertices in complex *C*_*i*_, and |∪*C*_*i*_| is the total number of vertices in Cset.

As shown in Table 1, the number of identified complexes is decreasing and the average size of identified complexes is increasing quickly with the increase of *λ*_th value. The possible reason is the larger value of *λ*_th which lead to more nodes added into the cluster when it is expanding and results in larger size of identified complex. Meanwhile, when a seed is expanding to a larger cluster with *λ*_th increasing, more other seeds are included in the cluster and deleted from the seed queue, which results in the decrease of the number of identified complexes. Table 1 shows that the average density of identified complexes is high for each *λ*_th value. It is because when a cluster is expanding, the node added into it every time is the node with the highest cluster property to the cluster. So, MCSE is also a density-based local search method and the protein complexes identified by it trend to dense subgraphs. However, as shown in Table 1, the minimum density of identified complexes in each output set is small, which means that unlike other methods based on dense subgraphs, such as CMC and MCSE, can also identify protein complexes with small density. The overlapping rates of all identified complex sets are more than 1, which means MCSE can identify overlapping protein complexes.

**Figure 2:**
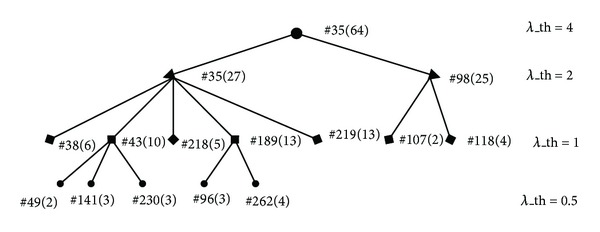
An example of hierarchical protein complexes identified by MCSE with different values of parameter *λ*_th.

When a seed is expanding, more nodes will be added with *λ*_th increasing, which causes the identified protein complexes in the set of lager *λ*_th value to include (fully or partially) those in the set of smaller *λ*_th value. So, by tuning *λ*_th value, MCSE can identify protein complexes in different levels. For example, the seven output sets in the Table 1 are composed of a hierarchical organization of protein complexes and Figure 2 illustrates part of it.

As shown in Figure 2, the identified protein complex #35 in the layer of *λ*_th = 4 includes two identified protein complexes #35 and #98 in the layer of *λ*_th = 2. Its sub-complex #35 in the layer of *λ*_th = 2 includes five identified protein complexes, #38, #43, #189, #218, and #219, in the layer of *λ*_th = 1. Another subcomplex #98 in the layer of *λ*_th = 2 includes two identified protein complexes, #107 and #118, in the layer of *λ*_th = 1. The identified protein complex #43 in the layer of *λ*_th = 1, which is a subcomplex of complex #35 in the layer of *λ*_th = 2, also includes three identified protein complexes, #49, #141, and #230, in the layer of *λ*_th = 0.5. Another subcomplex #189 in the layer of *λ*_th = 1 includes two identified protein complexes, #96 and #262 in the layer of *λ*_th = 0.5.

### 3.3. Comparison with Hierarchical Complexes in MIPS Database

**Figure 3:**
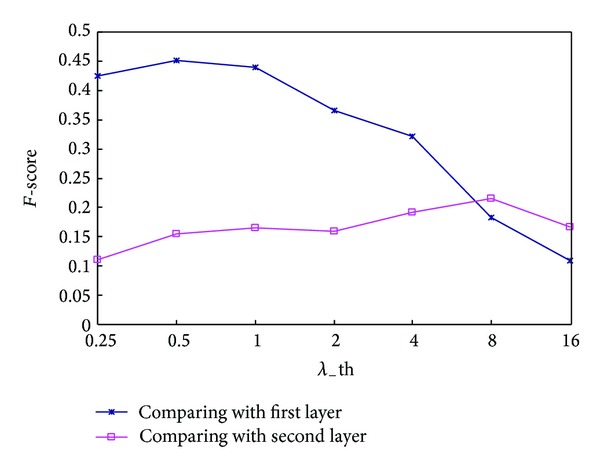
*F-score* of the complex sets identified by MCSE with different *λ*_th values with respect to overlapping scores threshold of 0.2 (Compared with the first and second layers of known hierarchical protein complexes, resp.).

Gavin and Krogan [35, 36] pointed out that some protein complexes are hierarchically organized and composed of several subcomplexes. To judge whether the hierarchical organization of complexes identified by MCSE is similar to that of known protein complexes of* S. cerevisiae* in MIPS database, we compare seven identified complex sets corresponding to different *λ*_th values with the first layer and second layers of known hierarchical complexes in MIPS database and list their* F-score* values in Figure 3.* F-score* values corresponding to the first layer form the blue line named as “comparing with first layer” and those corresponding to the second layer form the red line named as “comparing with second layer.” Here, we use* F-score* because it combines both sensitivity and specificity. The overlapping scores threshold is set as 0.2 because in many literatures, an identified complex and a known complex are considered as a match if their overlapping score is not less than 0.2 [12, 19, 21].

Seen from the blue line, it is obvious when comparing with the first layer that the* F-score* values of identified complex sets in low layers (*λ*_th ≤ 1) are much higher than those in high layers (*λ*_th ≥ 4). It means the identified complex sets in low layers match the first layer of hierarchical protein complexes better than those in high layers. On the contrary, seen from the red line, the identified complex sets in high layers (*λ*_th = 4 or *λ*_th = 8) match the second layer better than those in low layers (*λ*_th ≤ 2).

Compared with these two lines, we can see that when *λ*_th ≤ 4 the* F-score* value in blue line is higher than that in red line, but when *λ*_th > 4 the opposite is the case. It means the identified complex set in the low layer matches the first layer of the hierarchical known complexes better than the second layer, but with the identified complex set in high layer the opposite is the case. Concluding the above, the hierarchical structure of complexes identified by MCSE is similar to that of known complexes in MIPS database.

To compare the performance of MCSE and other seven complex detection methods for identifying complexes in different levels, we compare their identified complex sets with the first and second layers of known hierarchical complexes in MIPS database, respectively. The parameter values of all algorithms are selected the optimum values. As HC-PIN, NFC, and MCSE can identify hierarchical protein complexes, their parameter values are different when compared with the different layers. For example, Figure 3 shows the complex set identified by MCSE matches the first layer best when *λ*_th = 0.5 and the second layer best when *λ*_th = 8. So, the values of parameter *λ*_th of MCSE are set as 0.5 and 8 when compared with the first layer and second layer, respectively. Similarly, the parameter values of NFC and HC-PIN can also be obtained by experimental results. Notably, the experimental results show that whether compared with the first layer or with the second layer, the optimum value of parameter *α* of NFC is always 1. The other five algorithms, CMC, Core-Attachment, CPM, Dpclus, and MCL, cannot identify protein complexes in different layers. Thus, whether compared with the first layer or with the second layer, their selected parameter values are always those recommended by the authors.

**Figure 4:**
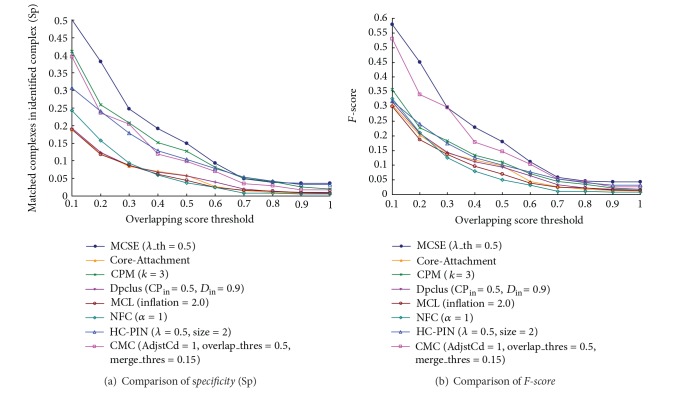
Compared with the first layer of known hierarchical protein complexes,* specificity* and* F-score* of MCSE and other algorithms.

Figure 4 list the values of* specificity* and* F-score* of MCSE and other seven algorithms when compared with the first layer. In the Figure, MCSE has the highest value of* specificity* and* F-score* in the eight algorithms for each overlapping score's threshold. For example, when overlapping score's threshold is the typical value of 0.2, the* specificity* value of MCSE is 0.38 and those of the other seven algorithms are from 0.12 to 0.26, which means the percentage of matched complexes in the complex set identified by MCSE is improved 48% to 223%. Meanwhile, the* F-score* value of MCSE is 0.45 and those of the other seven algorithms are from 0.19 to 0.34. Figure 4 shows that the protein complex set identified by MCSE matches the first layer of known hierarchical complexes in MIPS database better than other seven algorithms.

**Figure 5:**
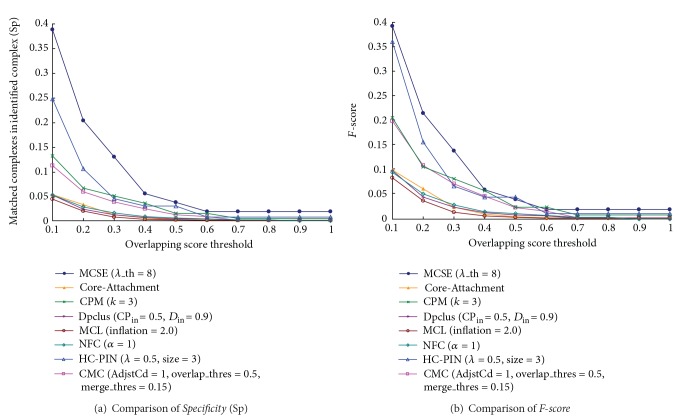
Compared with the second layer of known hierarchical protein complexes,* specificity* and* F-score* of MCSE and other algorithms.

Figure 5 list the values of* specificity* and* F-score* of MCSE and other seven algorithms when compared with the second layer. Figure 5 shows MCSE also has the highest values of* specificity* and* F-score*. It means the protein complex set identified by MCSE matches the second layer of known hierarchical complexes in MIPS database better than those identified by other seven algorithms. Notably, Figure 5 shows MCSE has much higher values of* specificity* and* F-score* when compared with the algorithms cannot identify hierarchical complexes. This is because MCSE can identify protein complexes in high layer by adjusting the value of parameter *λ*_th. Concluding the above, MCSE can identify protein complexes in different layers. So its identified complexes match the protein complexes in both low and high layers well.

### 3.4. Comparison with Other Algorithms in Terms of Matching with Known Complexes

**Figure 6:**
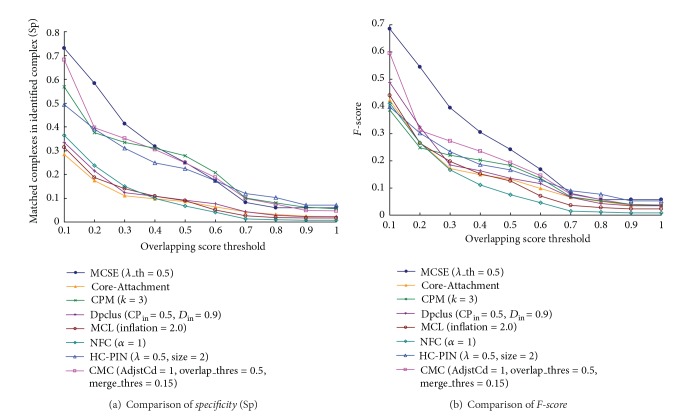
Compared with the latest known protein complexes of* S. cerevisiae*,* specificity* and* F-score* of MCSE and other algorithms.

To directly validate the effectiveness of algorithm MCSE for identifying protein complexes, we compare the protein complexes identified by MCSE and other seven algorithms with the latest known protein complexes of* S. cerevisiae* which provided in [40] and list their* specificity* and* F-score* in Figure 6, respectively. The known protein complex set used here is composed of leaf-complexes. So, the output set of MCSE should be the low layer and we set the parameter value of *λ*_th as 0.5.

As shown in Figure 6(a), when overlapping score's threshold is equal to 0.2, the* specificity* value of MCSE is 0.58, which means about 58% complexes detected by MCSE are matched by the known complexes. Compared with other seven methods, this ratio is improved 48% (compared with CMC) to 236% (compared with Core-Attachment) at the same threshold. Furthermore, Figure 6(a) shows that when overlapping score's threshold less than 0.5, the* specificity* value of MCSE is higher than those of other seven methods.

As shown in Figure 6(b), for each overlapping score's threshold, the* F-score* value of MCSE is higher than those of other seven methods (expect for those of HC-PIN when overlapping score's threshold is equal to 0.7 and 0.8), especially when overlapping score's threshold is not more than 0.6. For example, when overlapping score's threshold is equal to 0.2, the* F-score* value of MCSE is 0.54. Compared with the highest* F-score* value of the seven other algorithms (which is 0.32 of Dpclus), 68% improvement is obtained by using MCSE algorithm.

Figure 6(a) shows that the percentage of matched complexes in the complex set identified by MCSE is much higher than those identified by other seven methods. Figure 6(b) shows MCSE outperforms other seven methods by considering both* specificity* and* sensitivity*. All these indicate that our method MCSE identifies known protein complexes more effectively than other seven methods.

### 3.5. Comparison with Other Algorithms in Terms of Functional Enrichment

To evaluate the biological significance of complexes identified by MCSE, we calculate *P* value of each complex identified by MCSE and other seven methods in* YDIP*.  Table 2 lists the percentages of the identified complexes whose *P* value falls within *P*  value < *E* − 10, [*E* − 10, *E* − 5], [*E* − 5, 0.01], and ≥0.01. Generally speaking, an identified complex with *P* value less than 0.01 is considered significant [21, 27–29]. As shown in Table 2, 79.3% of complexes identified by MCSE are significant. Compared with the results of other seven methods, this percentage is improved, 22.6% (compared with CMC) to 122.1% (compared with Core-Attachment). On the other hand, the percentage of insignificant complexes identified by MCSE is not more than half of those identified by other seven methods. All these indicate the protein complexes identified by MCSE are more biologically significant than those identified by other seven methods.

### 3.6. The Effect of Seed Selection

MCSE is a “seed-expanding” method. To select seeds, we build a weighted PPI network* YDIP* ^*W*^ from* YDIP* according to formula (2) and choose seeds as PPIs whose weights are not less than the average weight of* YDIP* ^*W*^ and essential PPIs. The basic idea of this seed selection is the two kinds of PPIs are much more likely to be in a protein complex than those selected randomly and they are well complementary to each other.

To test the effect of this seed selection on identification of protein complexes, we compare it with other three strategies of seed selection. The first one selects seeds as PPIs whose weights are not less than the average weight of* YDIP* ^*W*^. The second one selects seeds as all essential PPIs. The third one selects seeds randomly and the number of these seeds is as same as that of MCSE. The modified MCSE algorithms based on the three strategies are named as MCSE (ECV), MCSE (Essential), and MCSE (Random), respectively.

**Figure 7:**
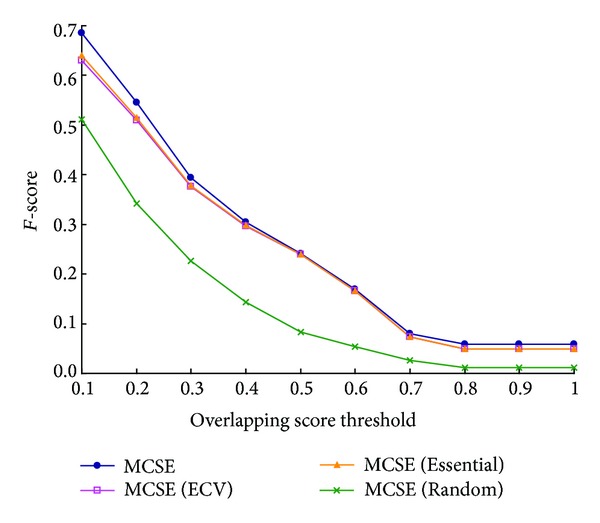
Comparison of* F-score* of MCSE and MCSE (ECV), MCSE(Essential), and MCSE(Random).

The protein complexes identified by MCSE and these three modified algorithms are compared with the latest known protein complexes of* S. cerevisiae* and their* F-score* are shown in Figure 7. The values of parameter *λ*_th of the four algorithms are set as 0.5. As shown in Figure 7, for each overlapping score's threshold, the values of* F-score* of MCSE (ECV) and MCSE (essential) are almost same and both much higher than that of MCSE (random). For example, when overlapping score's threshold is equal to 0.2, the values of* F-score* of MCSE (ECV) and MCSE (essential) are 0.509 and 0.515, respectively, and that of MCSE (random) is only 0.341. Compared with the value of* F-score* of MCSE (random), the values of* F-score* of MCSE (ECV) and MCSE (essential) are improved about 50%. It means seed selection is important for the performance of our method MCSE, and both MCSE (ECV) and MCSE (essential) are good seed selections. The reason is a PPI's essentiality and its edge clustering value in a PPI network are all effective factors to predict whether the PPI is in a protein complex or not. So, choosing seeds according to either of them can improve the performance.

As shown in Figure 7, for each overlapping score's threshold, the value of* F-score* of MCSE is highest. For example, when overlapping score's threshold is equal to 0.2, the value of* F-score* of MCSE is 0.545, which is improved 7% and 6% when compared with that of MCSE (ECV) and MCSE(essential), respectively. It means the performance can be improved further when combing both kinds of seeds. The reason is some protein complexes including PPIs with high edge clustering value but not essential PPIs, but with other protein complexes the opposite is the case. Obviously, the former kind of protein complexes cannot be identified by MCSE (essential) and the latter kind of protein complexes cannot be identified by MCSE(ECV). However, both kinds of protein complexes can be identified by MCSE.

### 3.7. The Effect of Weighted PPI Network

**Figure 8:**
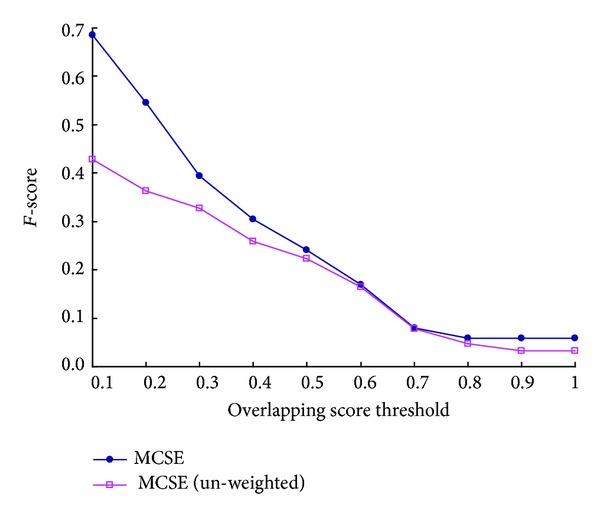
Comparison of* F-score* of MCSE and MCSE (un-weighted).

To improve the accuracy for identifying protein complexes, our method MCSE adopts two ways, selecting seed and building weighted PPI network. The effect of seed selection is discussed in the previous section. In this section, to discuss the effect of weighted PPI network, we modify algorithm MCSE as MCSE (unweighted) by expanding seeds on the unweighted PPI network* YDIP* instead of on the weighted PPI network* YDIP* ^*W*^ and compare the values of* F-score* of MCSE and MCSE (unweighted) in Figure 8. Here, the seed queues of both algorithms are same, the values of parameter *λ*_th of both algorithms are set as 0.5, and the known protein complexes are provided by [40].

As shown in Figure 8, for each overlapping score's threshold, the* F-score* value of MCSE is higher than that of MCSE (unweighted). For example, when overlapping score's threshold is equal to 0.2, the value of* F-score* of MCSE is 0.545 and that of MCSE (unweighted) is 0.362. The improvement of MCSE is 50.3%. It means the accuracy of MCSE for identifying protein complex can be improved effectively by expanding seeds on our weighted PPI network.

## 4. Conclusion

In the postgenome era, one major work is to identify protein complexes from large PPI networks. Various evidences have demonstrated they are overlapping and hierarchically organized [8–14]. However, it is still a challenge to identify hierarchical and overlapping protein complexes accurately in large PPI networks. Aiming at it, a novel method, namely, MCSE, is developed based on “seed-expanding” and *λ*-module. It is a local search algorithm and can identify protein complexes in a large PPI network quickly. As a protein can be added into several clusters when they are expanding from different seeds, MCSE can identify overlapping protein complexes naturally. Meanwhile, MCSE can detect hierarchical organization of overlapping protein complexes by tuning the value of parameter *λ*_th to control the expanding degree. Experimental results of* S. cerevisiae* show this hierarchical organization is similar to that of known protein complexes in MIPS database. We also compare the performances of our algorithm MCSE to other seven competing algorithms: CPM, CMC, Core-Attachment, MCL, Dpclus, NFC, and HC-PIN. Experimental results of* S. cerevisiae* show that our method MCSE outperforms them in terms of matching with known protein complexes and functional enrichment.

## Figures and Tables

**Algorithm 1:**
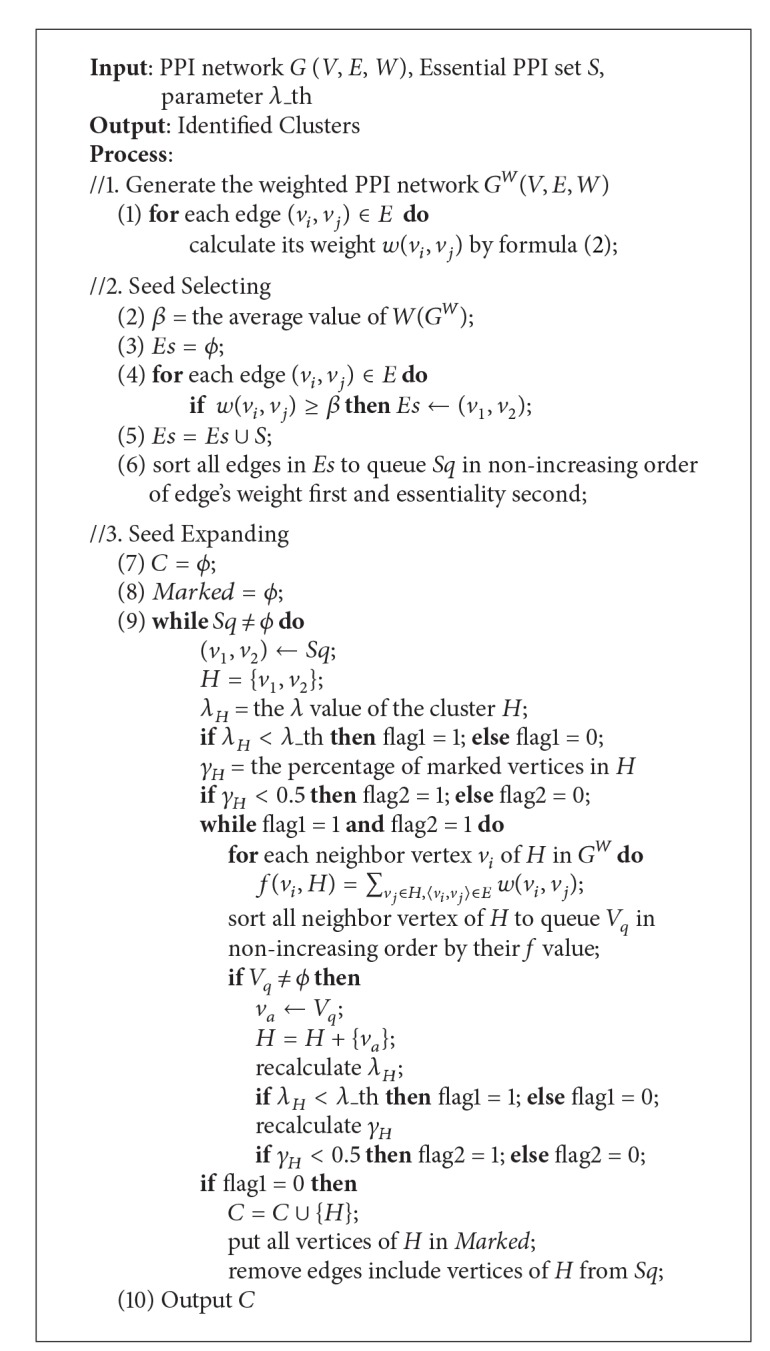
The description of algorithm MCSE.

**Table 1:** The effect of varying *λ*_th on clustering.

*λ*_th	Number	Average size	Average density	Minimum density	Overlapping rate
0.25	502	2.43	0.97	0.33	1.13
0.5	387	2.96	0.94	0.31	1.17
1	262	4.08	0.89	0.24	1.18
2	156	6.03	0.83	0.22	1.16
4	102	9.14	0.76	0.17	1.19
8	54	17.59	0.74	0.05	1.16
16	25	45.68	0.78	0.01	1.02

**Table 2:** Comparing the functional enrichment of protein complexes identified by MCSE and the seven other algorithms.

Algorithms	<*E* − 10	[*E* − 10, *E* − 5]	[*E* − 5, 0.01]	≥0.01 (insignificant)	<0.01 (significant)
MCSE (*λ*_th = 0.5)	7 (1.8%)	114 (29.5%)	186 (48.1%)	80 (20.7%)	307 (793%)
CMC (AdjstCD = 1, overlap_thres = 0.5, merge_thres = 0.15)	61 (8.4%)	131 (17.9%)	280 (38.4%)	258 (35.3%)	472 (64.7%)
Core-Attachment	76 (5.6%)	122 (9.0%)	287 (21.1%)	873 (64.3%)	485 (35.7%)
CPM (*k* = 3)	25 (12.7%)	49 (24.9%)	42 (21.3%)	81 (41.1%)	116 (58.9%)
Dpclus (CP_in_ = 0.5, *D*_in_ = 0.9)	42 (3.5%)	155 (12.9%)	329 (27.4%)	674 (56.2%)	526 (43.8%)
HC-PIN (*λ* = 0.5, size = 2)	40 (16.6%)	35 (14.5%)	84 (24.1%)	99 (55.2%)	166 (44.8%)
MCL (inflation = 2.0)	54 (5.8%)	114 (12.3%)	239 (25.7%)	522 (56.2%)	407 (43.8%)
NFC (*α* = 1)	47 (9.2%)	81 (15.6%)	124 (23.9%)	266 (51.3%)	252 (48.7%)
